# Atrial Arrhythmias in Patients with Severe COVID-19

**DOI:** 10.1155/2021/8874450

**Published:** 2021-03-12

**Authors:** Kai-Yue Han, Qi Qiao, Ye-Qian Zhu, Xin-Guang Chen, Xing-Xing Kang, Gao-Feng Zhang, Xun-Chao Cai, Yong Du, Jing Jin, Ruo-Min Di, Chen-Xi Yang, Feng-Xiang Zhang, Ying-Jia Xu

**Affiliations:** ^1^Department of Cardiology, The Fifth People's Hospital of Shanghai, Fudan University, Shanghai, China; ^2^Leishenshan Temporary Hospital, Wuhan, China; ^3^Department of Cardiology, The First Affiliated Hospital with Nanjing Medical University, Nanjing, China; ^4^Department of Bioinformatics, School of Medical Informatics and Engineering, Xuzhou Medical University, Xuzhou, China; ^5^College of Chemistry and Environmental Engineering, Shenzhen University, Shenzhen, China

## Abstract

The number of confirmed COVID-19 cases has increased drastically; however, information regarding the impact of this disease on the occurrence of arrhythmias is scarce. The aim of this study was to determine the impact of COVID-19 on arrhythmia occurrence. This prospective study included patients with COVID-19 treated at the Leishenshan Temporary Hospital of Wuhan City, China, from February 24 to April 5, 2020. Demographic, comorbidity, and arrhythmias data were collected from patients with COVID-19 (*n* = 84) and compared with control data from patients with bacterial pneumonia (*n* = 84) infection. Furthermore, comparisons were made between patients with severe and nonsevere COVID-19 and between older and younger patients. Compared with patients with bacterial pneumonia, those with COVID-19 had higher total, mean, and minimum heart rates (all *P* < 0.01). Patients with severe COVID-19 (severe and critical type diseases) developed more atrial arrhythmias compared with those with nonsevere symptoms. Plasma creatine kinase isoenzyme (CKMB) levels (*P*=0.01) were higher in the severe group than in the nonsevere group, and there were more deaths in the severe group than in the nonsevere group (6 (15%) vs. 3 (2.30%); *P*=0.05). Premature atrial contractions (PAC) and nonsustained atrial tachycardia (NSAT) were significantly positively correlated with plasma CKMB levels but not with high-sensitive cardiac troponin I or myoglobin levels. Our data demonstrate that COVID-19 patients have higher total, mean, and minimum heart rates compared with those with bacterial pneumonia. Patients with severe or critical disease had more frequent atrial arrhythmias (including PAC and AF) and higher CKMB levels and mortality than those with nonsevere symptoms.

## 1. Introduction

Novel coronavirus disease 2019 (COVID-19) is a newly recognized illness that has spread rapidly from Wuhan (Hubei province) to other areas in China and around the world [1, 2] and is considered one of the most serious threats to global health [3]. High-throughput sequencing from lower respiratory tract samples revealed a novel beta-coronavirus, preliminarily named 2019 novel coronavirus (2019-nCoV) and subsequently officially termed SARS-CoV-2 [4]. Initially, reported cases were mainly in China at the outset; however, as the epidemic has spread, more than 200 countries outside of China had reported almost 7.80 million cases of infection because of person-to-person transmission up to June 14, 2020 [5].

Notably, accumulating evidence indicates that SARS-CoV-2 infection has more effects than the typical respiratory symptoms; it can also attack multiple organs, including the heart [6], liver [7], and kidney [8], causing multiorgan failure, thus aggravating the condition and affecting patient prognosis. Few studies have investigated the effects of SARS-CoV-2 infection on the heart. A single-center study led by Peng described the clinical characteristics of 138 cases of COVID-19 from Zhongnan Hospital affiliated with Wuhan University, among which 16.70% developed arrhythmias, rising to 44.40% of patients with severe COVID-19 symptoms [9]; however, this study did not describe patient heart rates or specific types of arrhythmia [10].

In this study, we conducted a prospective study to investigate heart rate (total, mean, and minimum) and arrhythmias, including premature atrial contractions (PAC), nonsustained atrial tachycardia (NSAT), atrial fibrillation (AF), premature ventricular contractions (PVC), nonsustained ventricular tachycardia (NSVT), sustained ventricular tachycardia (SVT), paroxysmal supraventricular tachycardia (PSVT), and atrioventricular block (AVB), in patients with COVID-19 or bacterial pneumonia, using a remote mobile electrocardiogram (ECG) monitoring system. Cardiomyocyte injury markers and mortality were also analyzed in patients with COVID-19.

## 2. Methods

### 2.1. Study Population

COVID-19 patients (*n* = 84) treated at the Leishenshan Temporary Hospital, Wuhan (China), were enrolled from February 24 to April 5, 2020. Based on the World Health Organization (WHO) interim guidance for COVID-19, cases with COVID-19 were confirmed by the positive viral nucleic acid test result from throat swab samples. Patients with bacterial pneumonia (*n* = 84) at the First Affiliated Hospital with Nanjing Medical University, Nanjing (China), from June 28, 2017 to January 17, 2020, served as controls. Patients not confirmed with SARS-CoV-2 infection and lacking clinical data were excluded. This study was developed following the Declaration of Helsinki and was approved by the local institutional review board.

### 2.2. Data Collection and Definitions

Informed consent was obtained from all patients. Patient clinical electronic medical records, nursing records, and laboratory findings, such as cardiomyocytes injury markers, were collected. Data on age, sex, weight, comorbidities (hypertension, diabetes, hyperlipidemia, coronary artery disease (CAD), cardiomyopathy, heart failure (HF), chronic obstructive pulmonary disease, and lung cancer, among others), symptoms (fever, cough, fatigue, myalgia, headache, dyspnea, and diarrhea), medications (*β*-blockers, angiotensin-converting enzyme inhibitors (ACEI), angiotensin receptor blockers (ARB), calcium antagonist, diuretics, antidiabetes agents, nitrates, statins, antiplatelet agents, and antiarrhythmic therapy), and cardiomyocyte injury markers (creatine kinase isoenzyme (CKMB), high-sensitive cardiac troponin I (hscTnI), and myoglobin) were collected and analyzed.

### 2.3. Clinical Classification

Patients were diagnosed according to the WHO interim guidance for COVID-19 [11]. In this study, patients were divided into nonsevere (including mild and normal type) and severe (including severe and critical type) groups as follows. Mild type: mild clinical symptoms and imaging showing no pneumonia. Normal type: symptoms of fever, respiratory tract inflammation, and imaging showing pneumonia. Severe type, meeting any of the following criteria: respiratory distress; respiratory rate ≥30 breaths/min in the resting state; mean oxygen saturation ≤93%; and arterial blood oxygen partial pressure (PaO_2_)/fraction of inspired oxygen concentration (FiO_2_) ≤300 mmHg. Critical type: respiratory failure requiring mechanical ventilation and shock and combined failure of other organs requiring intensive care unit monitoring and treatment.

### 2.4. Analysis of ECG Monitoring Data

ECG monitoring data were obtained from all included patients with COVID-19 and bacterial pneumonia. Data were analyzed collectively as supraventricular arrhythmias (PAC, NSAT, AF, and PSVT), ventricular arrhythmias (PVC and NSVT), and AVB. NSVT was defined as three or more consecutive beats arising below the atrioventricular node with an RR interval of <600 ms (>100 beats/min) and lasting ≤30 s [12]. SVT was defined as continuous VT for 30 s or which required an intervention for termination (such as cardioversion) [13].

### 2.5. Statistical Analysis

Descriptive data are expressed as mean (standard deviation) or median (interquartile range (IQR)) for continuous variables and number (%) for categorical variables. Differences between the bacterial pulmonary infection and COVID-19 infection groups were evaluated using a two-sample *t*-test or Wilcoxon rank-sum test, depending on whether data were parametric or nonparametric for continuous variables, and a Fisher's exact test for categorical variables. The Spearman correlation coefficient was used for correlation analysis. Statistical significance was considered as *P* < 0.05.

## 3. Results

### 3.1. Clinical Characteristics

COVID-19 patients were of similar age and sex and had comparable comorbidities (hypertension, CAD, HF, stroke, liver disease, and kidney disease) to patients with bacterial pneumonia. COVID-19 patients had significantly lower rates of thyroid disease. Symptoms such as fever, myalgia, fatigue, and headache were more common in the COVID-19 group than in the bacterial pneumonia group (Table 1).

### 3.2. Heart Rate and Incidence of Arrhythmias in Patients with Bacterial Pneumonia and COVID-19

During hospitalization, compared with patients with bacterial pneumonia, those with COVID-19 had higher total, mean, and minimum heart rates (all *P* < 0.01) after standardization for ECG monitoring time. There was no significant difference in the incidence of arrhythmias, including PAC, NSAT, AF, PVC, NSVT, and AVB between the COVID-19 and bacterial pneumonia groups (all *P* > 0.05) (Table 2).

### 3.3. Comparison of Arrhythmias, Cardiomyocyte Injury Markers, and Death Rates between Patients with Nonsevere and Severe COVID-19

Comorbidities were similar between the two groups. During hospitalization, compared with those with nonsevere disease, patients with severe symptoms more frequently developed atrial arrhythmias, including PAC (median (IQR), 95.40 [6.10, 1410.40] vs. 6.40 [0.80, 25.60]; *P*=0.006) and AF (10% vs. 0; *P*=0.05); however, there were no significant differences in the incidence of NSAT, PVC, NSVT, or AVB (all *P* > 0.05). CKMB (*P*=0.004) and hscTnI (*P*=0.01) levels were higher in the severe group than in the nonsevere group, while myoglobin levels did not differ between them. There were more deaths in the severe group than in the nonsevere group (15% vs. 2.30%; *P*=0.05) (Table 3). Furthermore, there were correlations between PAC and NSAT and plasma CKMB levels, but not hscTnI and myoglobin levels (Table 4).

### 3.4. Comparison of Arrhythmias, Cardiomyocyte Injury Markers, and Death between <65-Year-Old and ≥65-Year-Old Patients with COVID-19

Older adults are more susceptible to developing critical illness, with higher resultant mortality. Therefore, we divided COVID-19 patients into two groups according to their age: <65 years old and ≥65 years old. There were no significant differences in the incidence of arrhythmias between the two groups (*P* > 0.05). Plasma CKMB (*P*=0.001) and hscTnI (*P*=0.01) levels were higher in the ≥65-year-old group than in the <65-year-old group; however, myoglobin levels did not differ between the two groups (*P* = 0.10), and there was no significant difference in mortality rates between them (*P*=0.13) (Table 5).

## 4. Discussion

This study provides data on heart rates and arrhythmias in patients with COVID-19, showing that they have higher total, mean, and minimum heart rates compared with individuals with bacterial pneumonia. However, the incidence of arrhythmias did not differ significantly between the COVID-19 and bacterial pneumonia groups. Among the 84 patients with COVID-19, those with severe symptoms had higher rates of PAC, AF, CKMB, and mortality.

In this study, we used a remote mobile ECG monitoring system, which is highly appropriate for investigation of heart rate and arrhythmia incidence, as it is free from various limitations, including length of recording time and space, allowing comprehensive ECG monitoring of patients, as well as 24 h recording, data storage, real-time alarm systems, and remote transmission and reception of relevant information. These advantages assist patients and responsible physicians in conducting analysis and making judgments. Furthermore, this approach can be used to record the electrical activity of the heart for 1–30 days in patients in their natural state, thereby providing an important basis for the diagnosis of arrhythmias.

Bacterial pneumonias are relatively common and have been extensively studied; however, outbreaks of deadly coronaviruses have demonstrated the need for constant vigilance in the face of new emerging pathogens [14]. Rabaan et al. reported that the SARS-CoV-2 and SARS-CoV genome sequences exhibit 79.50% homology [15]. The route of entry of SARS-CoV-2 into host cells is the same as that of SARS-CoV, that is, through angiotensin-converting enzyme 2 (ACE2) [16]. Furthermore, the common symptoms of SARS-CoV infection, including fever, dry cough, and fatigue, followed by anorexia, myalgia, and dyspnea, are also clinical characteristics in the majority of patients presenting with COVID-19 [17].

In this study, the result shows that the patients with COVID-19 had higher heart rate and/or more PAC/AF burden than individuals with bacterial pneumonia, which is first reported until now. This may be attributable to the fact that patients with COVID-19 had higher fevers, which can induce sympathetic excitation. In addition, SARS-CoV-2 infection can involve the myocardial interstitial tissue, stimulating a higher heart rate, related to ECG abnormalities such as STT abnormalities, arrhythmias, QTc interval prolongation, and pathological *Q* waves which were associated with increased odds of ICU admission [18]. Furthermore, in the course of SARS treatment, the heart rates of patients with arrhythmia were continuously increased [19]. The main manifestation was sinus tachycardia, which is inconsistent with the symptoms of fever and hypoxemia. During convalescence, patients' heart rates remained rapid, suggesting that myocardial damage may be involved. The same mechanism leads to tachyarrhythmia during SARS-CoV-2 infection [20].

The current study demonstrates that patients with severe infection appear to be at elevated risk of PAC and AF, with PAC occurring in 65.50% of those with COVID-19. This further reiterates the possibility that the severity of the infection could be a trigger for AF. The exact mechanism of action through which COVID-19 could trigger the onset of AF remains to be fully clarified. One possibility is direct myocardial damage. ACE2 is a membrane bound aminopeptidase that has vital roles in the cardiovascular and immune systems [21]. SARS-CoV-2 and SARS-CoV share a number of similarities, including that they both attack host cells via ACE2. By dissecting the heart and its circulatory system in patients who died of SARS, researchers found that the SARS-CoV can infect the heart and its circulatory system, causing viral myocardial inflammatory changes [21]. Second, SARS-COV-2 may induce cell death by upregulating the expression of genes related to “positive regulation of cell death” and “cell response to external stimulation,” which may cause myocardial damage. Finally, hypoxemia, fever, high level of proinflammatory cytokines, and proarrhythmic medication may also play a role in myocardial damage related to SARS-COV-2 infection [22].

However, it was noted that beyond the related COVID-19 mechanisms of arrhythmias, no difference was found with bacterial pneumonia in terms of incidence in this study. It is important to emphasize that the clinical symptoms of AF and other supraventricular diseases are generally caused by severe sepsis. A systematic review also reported a higher risk of AF, according to increasing grade of sepsis, in critically ill patients [23]. The mechanism of the increased incidence of atrial arrhythmia in patients with sepsis has not been fully elucidated. According to the reports, it may involve a variety of pathophysiological pathways caused by arrhythmogenic substrates, triggering factors, and regulatory factors [24]. For example, dystonia of the autonomic nervous system [25, 26], high catecholamine levels [27], and increased intracellular calcium are all the factors that trigger arrhythmias [28]. Based on the results in this study, although further research is needed to determine whether patients with COVID-19 also result in sepsis depending on these mechanisms, we suggest that patients with COVID-19 had arrhythmias due to the sepsis induced by hypoxia and acidosis.

Myocardial injury is an important contributor to fatal outcomes in patients with COVID-19, and in our study, plasma CKMB levels and mortality were higher in patients with severe COVID-19 than nonsevere COVID-19. CKMB primarily localizes to the outer plasma layer of cardiomyocytes and is the most specific enzyme in the myocardial enzyme spectrum used for clinical diagnosis of myocardial injury. Recently, numerous clinical studies have found that some patients with COVID-19 present with myocardial injury, with levels of plasma myocardial enzymes and cardiomyocyte injury markers increased by varying degrees [29, 30]. The risk of myocardial injury is reported to be higher in patients with severe disease [30, 31], consistent with our results. Moreover, we identified correlations between PAC and NSAT, with plasma CKMB levels, but not hscTnI and myoglobin levels.

## 5. Study Limitations

The main limitations of our study were as follows: first, the sample size was relatively small; however, 84 patients with COVID-19 were investigated, and the resulting data provide a profile of arrhythmias in this group of patients. Second, control patients with bacterial pneumonia patients were enrolled at a different hospital from study group patients. As COVID-19 is a highly contagious epidemic disease, control bacterial pneumonia patients could not be enrolled from the same hospital. Third, in this study, there was no significant difference regarding the incidence of atrial arrhythmias between the patients with COVID-19 and bacterial pneumonia. This may be the reason that supraventricular arrhythmias are better framed in the severity of the clinical picture, such as sepsis, rather than expression of COVID-19. In fact, during the course of treatment, we also found that a large number of COVID-19 patients had clinical symptoms of sepsis. Unfortunately, we did not take that into account.

## 6. Conclusions

In conclusion, this study found that patients with COVID-19 had higher total, mean, and minimum heart rate values than those with bacterial pneumonia. Patients with severe or critical disease more frequently developed atrial arrhythmias (including PAC and AF) and had higher CKMB levels and mortality rates.

## Figures and Tables

**Table 1:** Baseline characteristics of patients with bacterial pneumonia and COVID-19 patients.

Variable	Bacterial pneumonia (*N* = 84)	COVID-19 patients (*N* = 84)	*P* value
Age (yrs)	61.60 ± 11.30	61.90 ± 13.90	0.88
Male/female (*n*)	37/47	44/40	0.28
Weight (kg)	68.10 ± 22.60	66.70 ± 13.90	0.72
Hypertension (*n*)	30 (35.70)	34 (40.50)	0.53
Diabetes (*n*)	9 (10.70)	19 (22.60)	0.04
Hyperlipidemia (*n*)	2 (2.40)	2 (2.40)	1.00
CAD (*n*)	10 (11.90)	8 (9.50)	0.62
Thyroid disease (*n*)	10 (11.90)	1 (1.20)	0.01
Cardiomyopathy (*n*)	1 (1.20)	0	1.00
HF (*n*)	8 (9.50)	5 (6.00)	0.39
Stroke (*n*)	4 (4.80)	9 (10.70)	0.15
COPD (*n*)	10 (11.90)	3 (3.60)	0.04
Lung cancer (*n*)	19 (22.60)	1 (1.20)	<0.01
Liver disease (*n*)	2 (2.40)	7 (8.30)	0.17
Kidney disease (*n*)	0	4 (4.80)	0.12
Fever (*n*)	14 (16.70)	60 (71.40)	<0.01
Cough (*n*)	66 (78.60)	42 (50.00)	<0.01
Dyspnea (*n*)	14 (16.70)	22 (26.20)	0.13
Myalgia (*n*)	0	17 (20.20)	<0.01
Fatigue (*n*)	4 (4.80)	31 (36.90)	<0.01
Headache (*n*)	2 (2.40)	8 (9.50)	0.05
SBP (mm/Hg)	126 (114, 139)	130 (120, 139)	0.21
DBP (mm/Hg)	75 (69, 82)	80 (73, 87)	<0.01
Diarrhea (*n*)	0	3 (3.60)	0.25
*β*-Blockers (*n*)	7 (8.30)	18 (21.40)	0.02
ACEI/ARB (*n*)	11 (13.10)	7 (8.30)	0.32
Calcium antagonist (*n*)	13 (15.50)	20 (23.80)	0.17
Diuretics (*n*)	6 (7.10)	7 (8.30)	0.77
Antidiabetes agents (*n*)	6 (7.10)	11 (13.10)	0.20
Nitrates (*n*)	3 (3.60)	4 (4.80)	1.00
Statins (*n*)	16 (19.00)	11 (13.10)	0.29
Antiplatelet agents (*n*)	11 (13.10)	10 (11.90)	0.82
AADs (*n*)	0	13 (15.50)	<0.01
Liver-protecting agents (*n*)	8 (9.50)	11 (13.10)	0.47

Data are expressed as mean (SD) or median (IQR) for continuous variables and number (%) for categorical variables.

**Table 2:** Heart rate, arrhythmias, cardiomyocyte injury marker, and death in patients with bacterial pneumonia and COVID-19 patients.

Variable	Bacterial pneumonia (*N* = 84)	COVID-19 patients (nonstandardization) (*N* = 84)	COVID-19 patients (standardization) (*N* = 84)	*P* value^a^
Record time (*h*)	24	41.60 (25.90, 66.00)	24.00	<0.01
Total heart rate (beats)	100207 (92309.30, 11123)	208850 (128049.30, 302796.50)	115913.50 (108373.00, 129454.60)	<0.01
Maximum heart rate (bpm)	127.20 ± 22.70	129.50 ± 21.60	129.50 ± 21.60	0.73
Minimum heart rate (bpm)	48.30 ± 8.50	57.90 ± 10.50	57.90 ± 10.50	<0.01
Mean heart rate (bpm)	70.80 ± 10.70	82 ± 10.70	82 ± 10.70	<0.01
PAC (beats)	18 (4.50, 251.50)	30 (4, 329)	16.190 (2.70, 3943.40)	0.06
NSAT (*n*)	1 (0, 3.00)	0 (0, 3.80)	0 (0, 2.40)	0.14
AF (*n*)	3 (3.60)	4 (4.80)	4 (4.80)	1.00
PVC (beats)	2.5 (0, 35)	8 (1, 149)	4.39 (0.40, 91.40)	0.37
NSVT (*n*)	0 (0, 0)	0 (0, 0)	0 (0, 0)	0.30
SVT (*n*)	0	0	0	1.00
PSVT (*n*)	0	0	0	1.00
AVB (*n*)	0	3 (3.60)	3 (3.60)	0.25

Data are expressed as mean (SD) or median (IQR) for continuous variables and number (%) for categorical variables. AF, atrial fibrillation; AVB, atrioventricular block; COVID-19, novel coronavirus disease 2019; PAC, premature atrial contracts; PSVT, paroxysmal supraventricular tachycardia; PVC, premature ventricular contracts; NSAT, nonsustained atrial tachycardia; NSVT, nonsustained ventricular tachycardia; SVT, sustained ventricular tachycardia. ^a^Statistical differences between the COVID-19 patients (standardization) and COVID-19 patients (nonstandardization) groups.

**Table 3:** Arrhythmias, cardiomyocyte injury markers, and death between nonsevere and severe patients with COVID-19.

Variable	Nonsevere (*N* = 44)	Severe (*N* = 40)	*P* value
Male/female (*n*)	18/26	26/14	0.03
Age (yrs)	55.20 ± 11.50	69.30 ± 11.60	<0.01
Hypertension (*n*)	16 (36.40)	18 (45)	0.42
Diabetes (*n*)	8 (18.20)	11 (27.50)	0.31
Hyperlipidemia (*n*)	2 (4.50)	0	0.50
CAD (*n*)	2 (4.50)	6 (15)	0.14
Thyroid disease (*n*)	1(2.30)	0	1.00
Cardiomyopathy (*n*)	0	0	1.00
HF (*n*)	1 (2.30)	4 (10)	0.19
Stroke (*n*)	3 (6.80)	6 (15)	0.30
COPD (*n*)	0	3 (7.50)	0.10
Lung cancer (*n*)	1 (2.30)	0	1.00
Liver disease (*n*)	3 (6.80)	4 (10)	0.70
Kidney disease (*n*)	1 (2.30)	3 (7.50)	0.34
Total heart rate (beats/24 hrs)	115342.27 (108373.00, 127819.90)	118109.70 (110108.50, 136861.30)	0.28
Maximum heart rate (bpm)	131.40 ± 18.30	127.40 ± 24.70	0.75
Minimum heart rate (bpm)	58.70 ± 6.90	57.00 ± 13.50	0.46
Mean heart rate (bpm)	80.60 ± 7.70	83.50 ± 13.10	0.70
PCA (beats/24 hrs)	6.4 (0.80, 25.60)	95.40 (6.10, 1410.40)	<0.01
NSAT (*n*/24 hrs)	0 (0, 1.10)	1.10 (0, 29.40)	0.14
AF (*n*)	0	4 (10.00)	0.05
PVC (beats/24 hrs)	2 (0, 32.00)	8.3 (1.00, 202.10)	0.97
NSVT (*n*/24 hrs)	0 (0, 0)	0 (0, 0)	0.66
AVB (*n*/24 hrs)	0	3 (7.50)	0.10
CKMB (ng/mL)	0.9 (0.60, 1.40)	1.5 (1.20, 4.00)	<0.01
hscTnI (ng/mL)	0.01 (0.01, 0.01)	0.01 (0.01, 0.03)	0.01
Myoglobin (ng/mL)	4.8 (3.30, 11.20)	15.0 (6.90, 95.00)	0.07
Death (*n*)	1 (2.30)	6 (15.00)	0.05

Data are expressed as mean (SD) or median (IQR) for continuous variables and number (%) for categorical variables. AF, atrial fibrillation; AVB, atrioventricular block; CKMB, creatine kinase isoenzyme; COVID-19, novel coronavirus disease 2019; hscTnI, high-sensitive cardiac troponin I; PAC, premature atrial contracts; PVC, premature ventricular contracts; NSAT, nonsustained atrial tachycardia; NSVT, nonsustained ventricular tachycardia.

**Table 4:** The correlation between arrhythmias and cardiomyocyte injury markers (Spearman).

Variable (*N* = 84)	Correlation coefficient	*P* value
PAC		
CKMB	0.40	<0.01
hscTnI	0.20	0.08
Myoglobin	0.20	0.17
NSAT		
CKMB	0.40	<0.01
hscTnI	0.20	0.10
Myoglobin	0.10	0.28

CKMB, creatine kinase isoenzyme; hscTnI, high-sensitive cardiac troponin I; PAC, premature atrial contracts; NSAT, nonsustained atrial tachycardia.

**Table 5:** Arrhythmias, cardiomyocyte injury markers, and death between <65-year-old and ≥65-year-old patients with COVID-19.

Variable	<65-year-old (*N* = 48)	≥65-year-old (*N* = 36)	*P* value
Male/female (*n*)	25/23	19/17	0.95
Age (years)	52.30 ± 9.10	74.60 ± 7.20	<0.01
Total heart rate (beats/24 hrs)	6146624.13 (3979387.20, 9102121.30)	3788754.79 (2126971.80, 7602033.70)	0.94
Maximum heart rate (bpm)	133.70 ± 15.90	123.90 ± 26.60	0.12
Minimum heart rate (bpm)	59.20 ± 8.10	56.10 ± 13.00	0.39
Mean heart rate (bpm)	83.00 ± 8.80	80.60 ± 12.80	0.59
PAC (beats/24 hrs)	210.80 (30.30, 1131.50)	1717.50 (207.70, 52761.20)	0.12
NSAT (*n*/24 hrs)	0 (0, 20.00)	42.7 (0, 800.10)	0.21
AF (*n*)	1 (2.10)	3(8.30)	0.31
PVC (beats/24 hrs)	48 (0, 555.10)	1195.50 (45.30, 5012.50)	0.71
NSVT (*n*/24 hrs)	0 (0, 0)	0 (0, 0)	0.42
AVB (*n*/24 hrs)	0	3 (8.30)	0.08
CKMB (ng/mL)	0.90 (0.60, 1.40)	1.50 (0.90, 3.60)	<0.01
hscTnI (ng/mL)	0.01(0.01, 0.01)	0.01 (0.01, 0.02)	<0.01
Myoglobin (ng/mL)	5.00 (3.40, 12.20)	8.3 (6.80, 53.10)	0.11
Death (*n*)	2 (4.20)	5 (13.90)	0.13

Data are expressed as mean (SD) or median (IQR) for continuous variables and number (%) for categorical variables. AF, atrial fibrillation; AVB, atrioventricular block; CKMB, creatine kinase isoenzyme; COVID-19, novel coronavirus disease 2019; hscTnI, high-sensitive cardiac troponin I; PAC, premature atrial contracts; PVC, premature ventricular contracts; NSAT, nonsustained atrial tachycardia; NSVT, nonsustained ventricular tachycardia.

## Data Availability

The data used to support the findings of this study are included within the article.
